# A Preliminary Study on Kinetic Analysis of Ground Reaction Force and Impulse During Gait in Patients With Total Hip Replacement and Implication for Rehabilitation

**DOI:** 10.1111/os.14276

**Published:** 2024-10-30

**Authors:** Yuting Zhao, Wasim Raza, Graham Arnold, Penghai Li, Weijie Wang

**Affiliations:** ^1^ Department of Orthopaedic and Trauma Surgery, Orthopaedics and Rehabilitation Technology Centre, Ninewells Hospital and Medical School University of Dundee Dundee UK; ^2^ Department of Rehabilitation Tangdu Hospital of Air Force Medical University Xi'an China; ^3^ Department of Biomedical Engineering, LEJ Campus NED University of Engineering and Technology Karachi Pakistan; ^4^ School of Integrated Circuit Science and Engineering Tianjin University of Technology Tianjin China

**Keywords:** 3D impulse, gait analysis, ground reaction force, loading response phase, phase duration, total hip replacement

## Abstract

**Background:**

There is little research done on ground reaction forces (GRF) in terms of 3D impulses after total hip replacement (THR). This study aimed to investigate the GRFs and 3D impulses in four sub‐phases of stance during gait in the patients undergoing THR.

**Methods:**

A total of 10 pain‐free THR patients and 10 healthy people were recruited. This is an observational and retrospective study. The gait data was collected between 2008 and 2014 and analyzed between 2020 and 2024. All the participants were included in the three‐dimensional gait analysis. Gait parameters, phase durations, GRFs and impulses' key values during gait were calculated in four sub‐phases of stance. Statistical comparisons were performed with generalized linear models including age, gender, body mass index and walking speed as interactive factors.

**Results:**

It is found that (1) cadence, walking speed, stride length and step width in THR group were significantly decreased in compared with control group; (2) the THR decreased loading response duration in operative side and pre‐swing duration in non‐operative side compared with the control group, but the THR's two sides have similar duration proportions in sub‐stance phases; (3) the THR group had lower GRFs than the control group in vertical direction but higher in the medial–lateral direction; (4) in operative side, the THR's impulses in loading response phase were lower than the control group in anterior–posterior direction, and (5) in non‐operative side, the THR's impulse in pre‐swing phase in anterior–posterior direction was higher than the control side.

**Conclusion:**

The THR group showed slower walking speeds than the control group. The reasons could be from the decreased impulse in loading response phase, the decreased 2nd peak of GRF and the decreased pre‐swing impulse in vertical direction in operative side. Clinicians are suggested to consider the information provided when designing relevant rehabilitation exercises on the related muscles and functions.

## Graphical abstract

The Ground reaction forces for anterior–posterior (left), medial–lateral (centre) and vertical (right) direction. Red: operative‐side, Blue: non‐operative‐side, Green: control right side, Yellow: control left side. This study aimed to investigate the GRFs and 3D impulses in four sub‐phases of stance during gait in the patients undergoing THR. Ten pain‐free THR patients and ten healthy people were recruited. Gait parameters, phase durations, GRFs and impulses' key values during gait were calculated in four different sub‐phases of stance. It was found that the THR decreased loading response duration in operative side and pre‐swing duration in non‐operative side in compared with the control group. but the THR's two sides have similar duration proportions in sub‐stance phases.

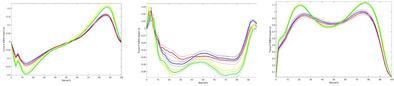

## 1 Introduction

The restoration of walking ability is part of what most total hip replacement (THR) patients expect [1], and the deficits in walking may lead to fatigue, thus affecting their ability to perform daily activities and reducing their quality of life [2]. However, 14%~22% of the patients with THR report various limitations of walking [3, 4]. In some recent literature, it is indicated that the postoperative gait of THR patients does not reach the same level as that of the general population, with reductions in terms of walking speed, stride length, sagittal hip, and the knee joint range of motion, as well as peak hip abduction [5, 6, 7]. Consequently, it may be less likely to achieve clinically meaningful improvements in functionality. Therefore, it is essential to prioritize the assessment of gait as a functional activity after THR. Quantitative gait analysis is required to better understand the gait mechanics which are potentially responsible for the functional restrictions observed in the patients who undergone surgery. Regarded as a standard tool for clinicians [8], ground reaction force (GRF) provides the information about basic locomotor mechanisms and the indirect information on internal joint loading through observation of the changes in various parameters [9, 10]. GRF impulse indicates the accumulation of force over time, reflecting not only the cumulative load over time in the three‐dimensional direction of the limb during gait [11], but also the change in velocity [12]. It shows a higher level of sensitivity to the quality of the gait than peak or average GRF [13].

The change in GRF after THR surgery has attracted the interest of research for decades, with many investigations conducted to reveal that atypical GRF persists after surgery. In as early as 1974, Stauffer [14] reported that the vertical peak forces were slightly lower for post‐surgery THR patients than for those in normal gait. In 2001, McCrory et al. [15] compared the GRF of the limbs on each side of the THR patients with the healthy subjects. However, the study focused only on the vertical direction and the demographic information about the two groups was inconsistent. In 2007, Bhargava et al. [16] collected the healthy subjects matched in age and sex for comparison with the patients in the THR group but ignored the effect of walking speed on GRF. Recently, most of the studies conducted on GRF impulse after THR are limited to the vertical direction, with only the sum impulses in an entire gait cycle as the focus [15, 17, 18, 19]. It thus remains unclear whether there are differences shown by the anterior–posterior and medial–lateral GRF, as well as 3D impulse changes in THR patients, from the healthy group during walking. Anterior–posterior GRF can reflect the braking and propulsive abilities. Reduced propulsion is thought to play a central role in reduced walking speeds. Medial–lateral GRF can reflect the walking stability and weight transfer. Therefore, it is necessary to explore whether the anterior–posterior and medial–lateral GFR and impulse of THR patients have changed.

In short, there is a lack of research on GRFs in THR in specific questions, for example, how the gait phases in THR are different from the control group, how the GRFs vary in different gait phases, how the GRFs change in 3D directions, and so forth. According to these questions, the hypotheses were that the THR gait phases would be different from the control group, for example, stance phase longer and swing one shorter; the GRFs of THR in the medial–lateral direction could change in a higher range than the control group. Therefore, the aim of this study was to investigate the characteristics of GRF and impulses in various phases of 3D gait between the patients with THR and those without THR, using Vicon motion capture system with force plates to collect subjects' 3D GRF and calculate impulses, and to further explore the differences between THR subjects and non THR subjects by dividing the stance phase into four sub‐phases. The results are expected to provide valuable information for clinicians to assess gait for THR patients and to support the rehabilitation programs carried out after THR surgery.

In structure, the paper is divided into a few of sections, (1) introduction of research background, questions and aim, (2) collection and analysis of gait data, (3) comparison of impulse and GRFs between THR and control groups by using tables and figures, (4) discussion on limitation and future studies, and (5) clinical relevance.

Regarding to specific research questions, this study had a few of scientific hypotheses as below. (1) THR's gait parameters might not be as good as those in the control group; (2) THR might have longer stance duration in operative side compared with the control group; (3) THR might have lower GRFs than the control group in all directions; (4) in operative side, the THR's impulses might be lower than the control group in some directions and some gait phases; and (5) in non‐operative side, the THR's impulse in some directions and some gait phases were higher than operative‐side and even higher than the control side. In the literature, there are not clear answers to these questions.

## 2 Materials and Methods

### 2.1 Participants

This study was approved by a Scotland NHS research ethics committee (09/S1401/65) and the University of Dundee the Research Ethical Committee (EB/MC/LET/LN 1384). All the participants in this study signed a consent form before data collection. The inclusion was that the THR patients (1) had completed surgery at least 1 year, (2) unilateral THR, (3) able to walk independently, and (4) pain‐free. The exclusion was that the THR ones (1) felt pain, (2) needed the walk aid, and (3) had their surgery within 1 year.

To reveal the effect of THR surgery on the long‐term gait of patients, those patients who received THR surgery within 1 year were excluded from this study. In total, 10 THR patients were included, including 3 males and 7 females, whose average age was 61.00 ± 8.26 years (range: 48–69 years). The average duration after THR was 1.80 ± 0.79 years (range: 1–3 years). To compare the surgical effects on gait, the data were divided into two categories: operative‐side and non‐operative‐side.

Ten participants without THR were included in the control group, including 7 males and 3 females, whose average age was 55.70 ± 2.67 years (range: 52–59 years). The inclusion criteria were as follows: (1) the participant didn't undergo surgery on the lower limbs or back previously and (2) the participant did not suffer from backache. The exclusion was (1) the age younger than 50 and (2) not sportsmen.

As the demographic data were slightly different between the two groups, the statistical analysis should include the factors, for example, gender, and height.

The demographic data including age, body mass, height and gender of the two groups is shown in Table 1.

**TABLE 1:** Demographic information of the two groups.

Measure	THR group	Control group	*T*/*χ* ^2^ values	*p*
	Mean ± SD	Mean ± SD		
	(min–max)	(min–max)		
Age (years)	61.00 ± 8.26 (48–69)	55.70 ± 2.67 (52–59)	−1.83	0.080
Body mass (kg)	80.40 ± 14.29 (55–98)	73.20 ± 7.49 (58–83)	−1.41	0.175
Height (m)	1.63 ± 0.49 (1.55–1.70)	1.71 ± 0.84 (1.59–1.84)	2.707	0.014*
Gender (male/female)	3/7	7/3	3.200	0.074

*Note: T*‐test for age, height, and body mass and Chi‐square test for gender were used.

*p < 0.05.

### 2.2 Equipment and Data Collection

The gait data was collected by using a Vicon motion capture system (Oxford, UK) equipped with 12 Vicon MX 40 cameras at sampling speed of 100 frames per second. The GRFs were measured using two Kistler 9281B force plates (Kistler Company, Switzerland) embedded within a laboratory floor surface and sampling speed 1000 HZ synchronized with the motion capture system. Vicon motion capture system combined with force platforms has been widely used in clinical gait analysis, and the validation of these instruments was done by previous studies [5, 19].

All the participants with 16 markers placed on their lower limbs on bony protuberance according to Vicon Clinical Management System and Plug‐in‐Gait model. They were required to walk with bare feet on a 20 m‐long walkway in the gait analysis laboratory. A minimum of 20 trials were conducted for each of them. Any trial with low‐quality data was excluded. The exclusion criteria are as follows: (1) the patient's foot wasn't rested entirely on the force plate; (2) the gait of the patient was unnatural, or the patient consciously adjusted the gait to ensure their foot on the plate; (3) the markers on the subjects were missing during walking. In total, 114 trials were included for further analysis.

After the valid trials were determined, the markers used for lower limbs were labeled manually.

To facilitate subsequent data analysis, gait cycle events were manually defined, including the heel strikes and toe off for each side. The heel strike and toe off on force platform was used for the definitions of events. After labelling, Static and Dynamic Plug‐in‐Gait models were applied to calculate the gait parameters, such as walking speed, stride length, and joint parameters.

### 2.3 GRF

GRF was measured in three dimensions. For vertical force (Fz), the magnitude of the first and second peaks (Fz1, Fz2) was calculated. For anterior–posterior force (Fx), the anterior was defined as positive and the posterior as negative, with the Fx positive peak force (Fxpp) and Fx negative peak force (Fxnp) measured. For medial–lateral force (Fy), the medial was defined as positive and the lateral was defined as negative, with the Fy positive peak force (Fypp) and Fy negative peak force (Fynp) calculated. All the GRF measures were normalized by BM or BW (body mass or body weight) and expressed as N/kg or N/BW.

### 2.4 Impulse

In classical mechanics, impulse (symbolized by) refers to the integral of a force (*F*) applied at the interval time (*t*) and is the change in momentum of an object.

The impulse (IP) is calculated by:

(1) $$I=\int \mathrm{Fdt}=mv_{2}-mv_{1}$$

(2) $$J=\sum_{t_{1}}^{t_{2}}\left({\mathrm{GRF}}_{i}-\mathrm{mg}\right)\mathrm{dt}$$

when *GRF* in vertical direction.

(3) $$J=\sum_{t_{1}}^{t_{2}}\left({\mathrm{GRF}}_{i}\right)\mathrm{dt}$$

when *GRF* in horizontal direction.

(4) $$\sum_{t_{1}}^{t_{2}}\left(\frac{{\mathrm{GRF}}_{i}}{m}-g\right)\mathrm{dt}=v_{2}-v_{1}$$

when *GRF* in vertical direction.

(5) $$\sum_{t_{1}}^{t_{2}}\left(\frac{{\mathrm{GRF}}_{i}}{m}\right)\mathrm{dt}=v_{2}-v_{1}$$

when *GRF* in horizontal direction.

where

- *F* is the resultant force applied from *t*_1_ to *t*_2_ and was GRF in this study.
- *t*_1_ and *t*_2_ are the instants when impulse begins and ends, respectively, and were gait phases or cycle times in this study.
- *m* is the mass of the subject and was the body mass in this study.
- *v*_1_ and *v*_2_ are the start and finish velocities in related gait phases or cycles.
- *dt* is the time interval between two frames of GRF, that is, 1/(GRF Hz).

The Equation (1) is from classical mechanics and the principle to calculate the impulses. In practice, the impulse was calculated by the sum of the products of GRFs and time interval as Equations ((2), (3), (4), (5)) and the beginning and ending instants were determined by a specific period of time, for example, sub‐stance phases, one by one. The stance phase was divided into four phases according to the GRF data. The loading response (LR) was defined that one side of foot initially strikes on the ground until the contralateral foot leaves from the ground; mid‐stance (MS) phase was defined that one side foot starts standing using a single foot until 50% of stance phase; terminal stance (TS) was defined that one foot maintains standing from 50% of stance phase until the contralateral foot strikes on the ground; and pre‐swing (PS) phase was defined that both feet on the ground until one side of foot leaves the ground [20]. In this study, the sub‐phase durations during stance were calculated according to these definitions.

**FIGURE 1:**
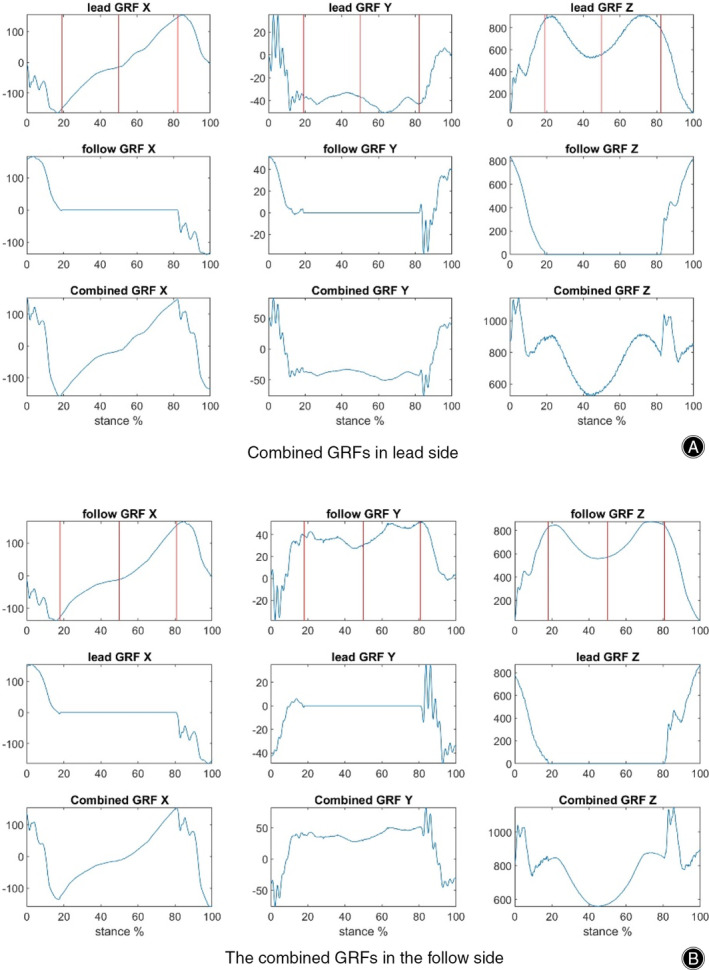
The combining GRFs from two sides of GRFs. Two sides were defined as the lead or follow sides where the first touched a force platform was lead side and the other was follow one. Three vertical red lines represent (1) the loading response finishing instant when the contralateral foot takes off the ground; (2) the 50% stance instant and (3) the pre‐swing starting instant when the contralateral foot strikes at the ground again. The instant events were determined by the GRFs and gait. X, Y, and Z represent the anterior–posterior, medial–lateral, and vertical directions.

To calculate the impulse, the GRFs from both sides were combined into the stance phase. This way responds to the real walking situation where both side legs coordinately work to contribute to the stance phase for whole body as Figure 1. The LR, MS, TS, and PS sub‐stance phases were also determined by the instants (red lines) in Figure 1 and were calculated as percentages of whole stance phase.

The sum impulses in three directions (xIP, yIP, and zIP, respectively) during these four phases were calculated, representing the cumulative load applied in three directions to the whole body in four different phases during the stance phase. In theory, the impulses can be calculated by integrating GRF with the small interval during stance. To simplify the calculation process, the impulse was calculated by summing GRFs with time interval in the system, that is, 1/1000 s from force platforms. The impulses were calculated in the four sub‐stance phases during stance in three directions.

### 2.5 Statistical Data Analysis

SPSS v 28 statistical analysis software was used in this study to conduct data analysis, with the data indicated as mean ± SD/SR (standard deviation or standard error of mean). The data distribution was examined by performing the Kolmogorov–Smirnov test. *T* test or Chi‐square test was conducted to examine the demographic data of the two groups. In this study, the participants were asked to walk at their comfortable speed instead of specifying the range of speed. In previous study, it has been illustrated that walking speed has a significant effect on GRF values [21]. To eliminate the potential impact of walking speed, a general line model for multivariate was applied to compare GRF peak forces and impulses between the two groups, with “group” as between‐ subject factors, “walking speed” as covariates. The multivariable model was constructed by introducing walking speed, age, gender, and BMI as interaction factors (i.e., the fixed and covariate ones) [22, 23, 24]. Putting the factors of age, gender, BMI, and walking speeds into the model made the statistical procedure able to reduce the effect of these factors to the outcomes, that is, the 3D GRFs and impulses. In practice, it is difficult to collect two groups with the identical background with these demographic and gait data. Therefore, it is necessary to use the statistical model to deal with these factors. Adjustment for multiple comparisons was Bonferroni. *p* < 0.05 was treated as statistically significant.

### 2.6 Research Path

**FIGURE 2:**
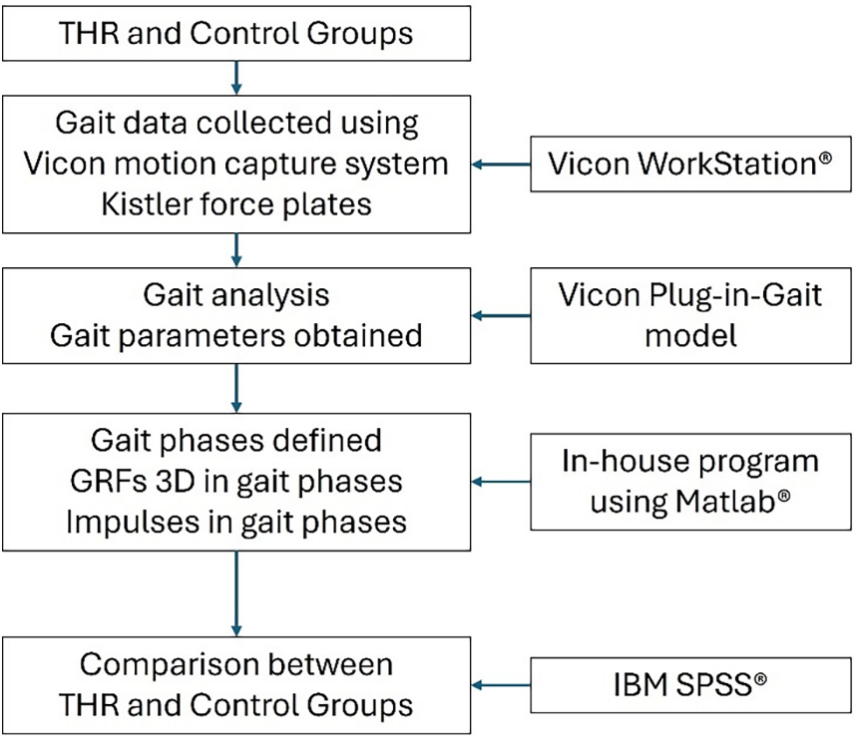
The research path of this project.

In summary, the research stages are described as in the flowing chart of Figure 2.

## 3 Results

### 3.1 Spatiotemporal Variables Comparison

According to the results, the magnitude of walking speed, step length, and cadence in control group was significantly greater than the THR group, as shown in Table 2. The duration percentages in different phases are shown in Tables 3 and 4.

**TABLE 2:** Comparison of basic gait variables in THR and control groups.

Dependent variable		Mean	Std. error	*p*
Cadence (steps/min)	Control	112.88	0.82	< 0.0001*
	THR	106.16	0.73	
Walking Speed (m/s)	Control	1.20	0.02	< 0.0001*
	THR	1.03	0.02	
Stance % gait cycle %	Control	61.29	0.34	0.076
	THR	62.17	0.30	
Stride Length (m)	Control	1.28	0.02	< 0.0001*
	THR	1.16	0.01	
Step Length (m)	Control	0.64	0.01	< 0.0001*
	THR	0.58	0.01	
Step width (m)	Control	0.18	0.001	< 0.0001*
	THR	0.15	0.001	

*Note*: Covariates appearing in the model are evaluated at the following values: BMI = 27.8833, age = 58.54. Adjustment for multiple comparisons: Bonferroni. Gender as fixed factor.

*p < 0.05.

**TABLE 3:** Comparison of sub‐phases percentages between the THR and control groups in stance phase.

Phase	Group	Mean	SR	*p*
LR	Control	20.77	0.62	0.004*
	THR‐OP	17.62	0.68	
MS	Control	29.23	0.62	0.004*
	THR‐OP	32.38	0.68	
TS	Control	31.86	0.47	0.611
	THR‐OP	32.26	0.51	
PS	Control	18.15	0.47	0.611
	THR‐OP	17.74	0.51	
LR	Control	18.07	0.51	0.909
	THR‐Non‐OP	17.97	0.56	
MS	Control	31.93	0.51	0.909
	THR‐Non‐OP	32.03	0.56	
TS	Control	29.33	0.61	0.008*
	THR‐Non‐OP	32.16	0.66	
PS	Control	20.67	0.61	0.008*
	THR‐Non‐OP	17.84	0.66	

*Note*: Covariates appearing in the model are evaluated at the following values: BMI = 27.88, age = 58.54, walking speed = 1.16. Adjustment for multiple comparisons: Bonferroni. Gender as a fixed factor.

Abbreviations: Control: the control group with the same side as the THR side; LR: loading response; MS: mid‐stance; PS: pre‐swing phases; THR‐OP or THR‐Non‐OP: the THR group's side with or without operation; TS: terminal stance.

*p < 0.05.

**TABLE 4:** Comparison of sub‐phases percentages between two sides in control and THR groups, respectively.

Group	Phase	Side	Mean	SR	*p*
Control	LR	Right	18.72	0.47	0.016*
		Left	17.37	0.54	
	MS	Right	31.28	0.47	0.016*
		Left	32.63	0.54	
	TS	Right	32.67	0.51	0.013*
		Left	31.26	0.50	
	PS	Right	17.33	0.51	0.013*
		Left	18.74	0.50	
THR	LR	OP	19.28	0.59	0.489
		Non‐OP	18.75	0.35	
	MS	OP	30.72	0.59	0.489
		Non‐OP	31.25	0.35	
	TS	OP	31.44	0.32	0.187
		Non‐OP	30.55	0.56	
	PS	OP	18.56	0.32	0.187
		Non‐OP	19.45	0.56	

*Note*: Control: Covariates appearing in the model are evaluated at the following values: BMI = 24.96, age = 55.69, walking speed = 1.29. THR: Covariates appearing in the model are evaluated at the following values: BMI = 30.79, age = 60.76, walking speed = 1.04. Adjustment for multiple comparisons: Bonferroni. Gender as a fixed factor.

Abbreviations: Control: the control group with the same side as the THR side; LR: loading response; MS: mid‐stance; PS: pre‐swing phases; TS: terminal stance.

*p < 0.05.

From Table 3, it is found that THR op‐side had shorter LR and longer MS durations than the control side, but in non‐operative side, THR group had shorter PS and longer TS durations than the control side. This finding meant that THR group decreased the LR and PS periods in different sides. However, THR group's both sides had similar phase durations, no matter of op‐ or non‐op side (Table 4). It indicated that THR group has a well recovery for their operative side after the surgery and the gait is symmetrical. Interestedly, the control group showed that both sides had different sub‐phases percentages (Table 4), which is worth being explored in the future.

### 3.2 Ground Reaction Force Comparison

**FIGURE 3:**
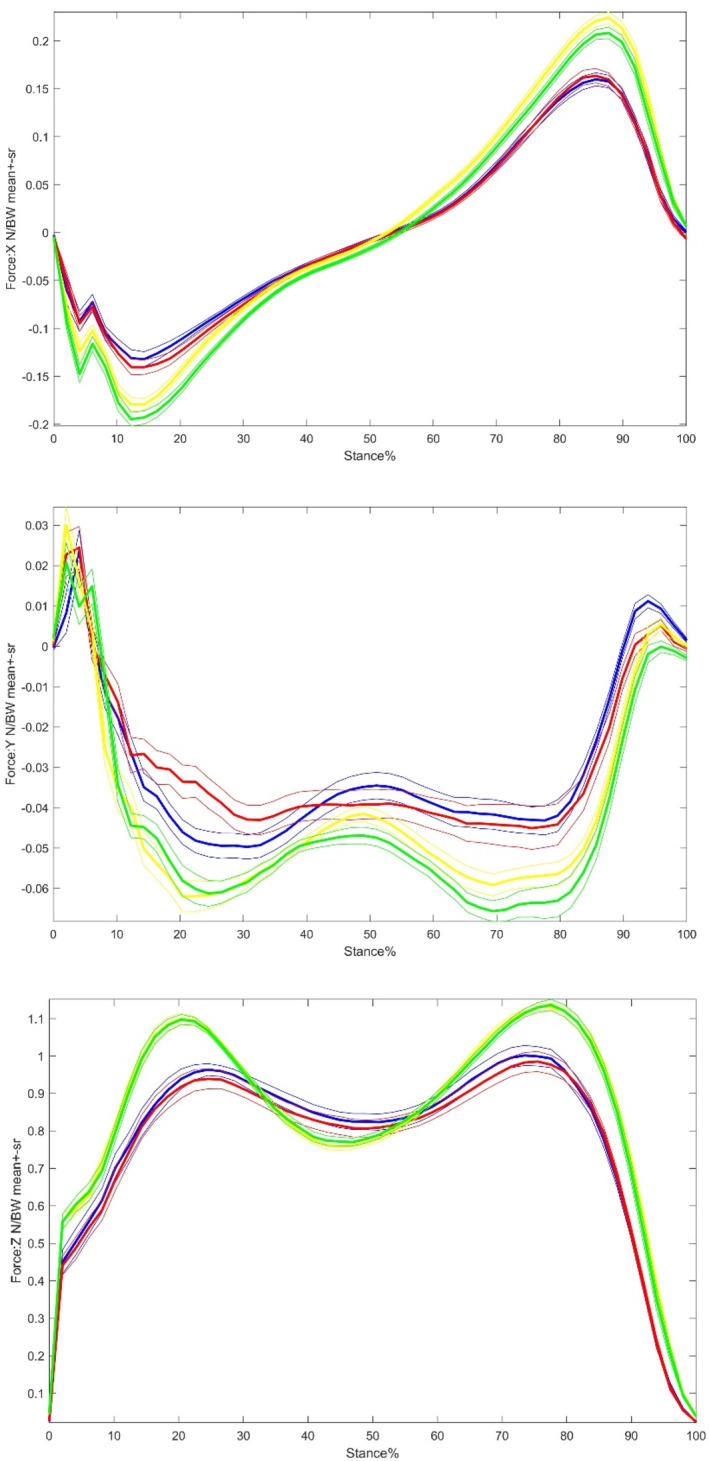
A time‐normalized traces of the ground reaction forces of control and THR groups during walking. Thickness lines are mean and thin lines ± 1 SR variability bands. Red: operative‐side and, Blue: non‐operative‐side, Green: control right side, Yellow: control left side. Upper: anterior–posterior direction; Middle: medial–lateral direction, and Lower: vertical direction. All curves are normalized by body weight.

Compared with the control group, the THR group showed a significantly lower level of GRF amplitudes in Fx, Fy, and Fz (Table 5 and Figure 3). As for the GRF amplitudes in Fz, only Fz2 varied significantly between the two groups, with no significant change observed in Fz1. Both Fxpp and Fynp were reduced to a significant extent on both sides of the THR group. However, only the Fypp on the operative side in THR group was significantly larger than the control group.

**TABLE 5:** Comparison of 3D ground reaction forces in THR group and control group.

Variable (N/BW)		Mean	Std. error	*p*
FXnp	Control	−0.18	0.01	0.125
	THR	−0.16	0.01	
FXpp	Control	0.20	0.00	0.528
	THR	0.20	0.00	
FYpp	Control	0.01	0.00	< 0.0001*
	THR	0.02	0.00	
FYnp	Control	−0.08	0.00	0.110
	THR	−0.07	0.00	
FZ1stpeak	Control	1.05	0.02	0.728
	THR	1.04	0.02	
FZ2ndpeak	Control	1.12	0.02	0.017*
	THR	1.04	0.02	

*Note*: Covariates appearing in the model are evaluated at the following values: BMI = 28.03, age = 58.36, walking speed = 1.16. Adjustment for multiple comparisons: Bonferroni. Gender as a fixed factor.

*p < 0.05.

Compared with the THR group, the control group had the value of Fypp as a half of the THR group (Table 6).

**TABLE 6:** Comparison of 3D ground reaction forces among operative‐ and non‐operative and control‐sides.

GRF (N/BW)		Mean	Std. error	Pair	*p*
FXnp	Control	−0.18	0.006		
	Op‐side	−0.161	0.008		
	Non‐Op	−0.166	0.008		
FXpp	Control	0.202	0.003		
	Op‐side	0.202	0.005		
	Non‐Op	0.195	0.005		
FYpp	Control	0.008	0.001	Con vs. Op	< 0.001*
	Op‐side	0.018	0.002		
	Non‐Op	0.018	0.002	Con vs. Non‐op	< 0.001*
FYnp	Control	−0.076	0.003		
	Op‐side	−0.069	0.004		
	Non‐Op	−0.066	0.004		
FZ 1st peak	Control	1.05	0.018		
	Op‐side	0.993	0.024	Op vs. Non‐op	0.006*
	Non‐Op	1.087	0.025		
FZ 2nd peak	Control	1.117	0.019	Con vs. Op	0.022
	Op‐side	1.022	0.025		
	Non‐Op	1.062	0.026		

*Note*: Covariates appearing in the model are evaluated at the following values: age = 58.36, BMI = 28.0299, walking speed = 1.16. Gender as a fixed factor. Adjustment for multiple comparisons: Bonferroni. Other pairs are with *p* > 0.05.

Abbreviations: Con: control; Non‐op: non‐operative side; Op: operative side.

*p < 0.05.

### 3.3 Impulse Comparison

#### 3.3.1 Operative Versus Control Sides

**FIGURE 4:**
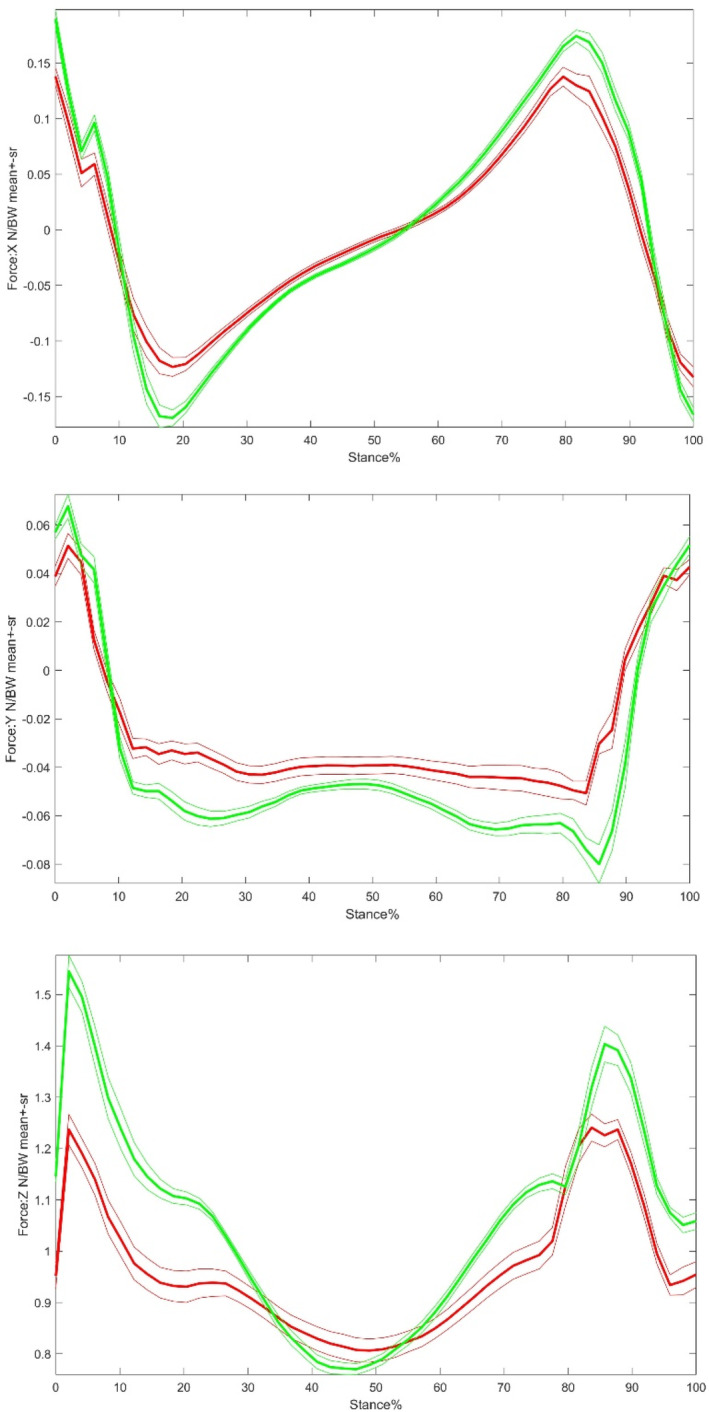
Comparison of combined GRFs from both sides in stance phase between the control group (green) and the THR operative side (red). X, Y, and Z represent the anterior–posterior, medial–lateral, and vertical directions. Green lines are the control and red the THR operative sides.

After both sides of GRFs are combined as in previous Figure 1, the GRFs in stance phase from the two groups are shown in Figure 4.

The magnitude of xIP in the loading response phase was substantially lower on the THR patients when compared with those in control group. In the yIP, the THR on pre‐swing phase was significantly larger than the control group, which meant that THR walking was not stable as the control group in the medial–lateral direction. Additionally, the zIPs in the THR group were significantly lower than in the control group during the loading response phases as shown in Tables 7 and 8. However, there was no significant difference found in 3D sum impulses between the two sides in THR group in whole cycle, as shown in Table 9.

**TABLE 7:** Comparison of impulses in all directions in all phases between the operative and control sides.

Direction	Phase		Mean	Std. error	*p*
X	WC IP	Control	0.003	0.002	
		OP	0.001	0.002	
	LR IP	Control	0.007	0.001	0.011*
		OP	0.002	0.001	
	MS IP	Control	−0.019	0.001	
		OP	−0.017	0.001	
	TS IP	Control	0.013	0.001	
		OP	0.014	0.001	
	PS IP	Control	0.002	0.001	
		OP	0.002	0.001	
Y	WC IP	Control	−0.024	0.002	
		OP	−0.019	0.002	
	LR IP	Control	0.003	0.000	
		OP	0.002	0.001	
	MS IP	Control	−0.012	0.001	
		OP	−0.01	0.001	
	TS IP	Control	−0.013	0.001	
		OP	−0.011	0.001	
	PS IP	Control	−0.002	0.000	0.016*
		OP	−0.0006	0.000	
Z	WC IP	Control	0.698	0.015	0.029*
		OP	0.64	0.017	
	LR IP	Control	0.146	0.004	< 0.0001*
		OP	0.117	0.005	
	MS IP	Control	0.208	0.006	
		OP	0.194	0.006	
	TS IP	Control	0.211	0.006	
		OP	0.2	0.006	
	PS IP	Control	0.138	0.003	
		OP	0.132	0.003	

*Note*: Covariates appearing in the model are evaluated at the following values: BMI = 28.03, age = 58.36, and walking speed = 1.16. Gender as a fixed factor. Adjustment for multiple comparisons: Bonferroni.

Abbreviations: Control: healthy group; IP unit: Ns/BW, and BW is body weight; IP: impulse; LR: loading response 0%–16% of stance; MS: middle stance, 16%–50% of stance; OP: operative side; PS: pre‐swing, 83%–100% of stance; TS: terminal stance, 50%–83% of stance; WC: whole cycle.

*p < 0.05.

**TABLE 8:** Comparison of impulses in all directions in all phases between the non‐operative and control sides.

Direction			Mean	Std. error	*p*
X	WC IP	Control	0.002	0.002	
		Non‐OP	0.006	0.002	
	LR IP	Control	0.005	0.001	
		Non‐OP	0.003	0.001	
	MS IP	Control	−0.018	0.001	0.013*
		Non‐OP	−0.015	0.001	
	TS IP	Control	0.016	0.001	0.006*
		Non‐OP	0.012	0.001	
	PS IP	Control	−0.001	0.001	0.002*
		Non‐OP	0.005	0.001	
Y	WC IP	Control	−0.02	0.002	
		Non‐OP	−0.019	0.002	
	LR IP	Control	0.003	0.000	
		Non‐OP	0.002	0.000	
	MS IP	Control	−0.011	0.001	
		Non‐OP	−0.011	0.001	
	TS IP	Control	−0.011	0.001	
		Non‐OP	−0.009	0.001	
	PS IP	Control	−9.40E‐05	0.000	
		Non‐OP	−1.70E‐05	0.000	
Z	WC IP	Control	0.716	0.015	0.019*
		Non‐OP	0.657	0.016	
	LR IP	Control	0.135	0.003	
		Non‐OP	0.127	0.003	
	MS IP	Control	0.212	0.004	
		Non‐OP	0.21	0.005	
	TS IP	Control	0.225	0.007	
		Non‐OP	0.208	0.007	
	PS IP	Control	0.149	0.004	< 0.0001*
		Non‐OP	0.114	0.004	

Abbreviations: Control: the control group with the same side as the THR side; LR: loading response; MS: mid‐stance; PS: pre‐swing phases; TS: terminal stance.

*p < 0.05.

**TABLE 9:** Comparison of impulses in all directions in all phases between the operative and non‐operative sides.

Group	Direction	Phase	Side	Mean	Std. error	*p*
Control^a^	X	WC	Left	0.053	0.018	
			Right	0.042	0.013	
		LR	Left	0.038	0.004	
			Right	0.039	0.008	
		MS	Left	−0.163	0.010	
			Right	−0.17	0.007	
		TS	Left	0.139	0.008	
			Right	0.134	0.009	
		PS	Left	0.041	0.008	
			Right	0.039	0.004	
	Y	WC	Left	0.209	0.010	< 0.0001**
			Right	−0.229	0.009	
		LR	Left	−0.02	0.003	< 0.0001**
			Right	0.019	0.003	
		MS	Left	0.114	0.005	< 0.0001**
			Right	−0.108	0.004	
		TS	Left	0.098	0.005	< 0.0001**
			Right	−0.121	0.005	
		PS	Left	0.019	0.003	< 0.0001**
			Right	−0.021	0.003	
	Z	WC	Left	0.51	0.040	
			Right	0.523	0.039	
		LR	Left	0.299	0.017	0.005*
			Right	0.421	0.038	
		MS	Left	−0.155	0.007	
			Right	−0.154	0.010	
		TS	Left	−0.058	0.012	
			Right	−0.045	0.015	
		PS	Left	0.423	0.038	0.005*
			Right	0.3	0.017	
THR^b^	X	WC	Non‐OP	0.018	0.016	
			OP	0.008	0.014	
		LR	Non‐OP	0.019	0.007	
			OP	0.017	0.008	
		MS	Non‐OP	−0.134	0.006	
			OP	−0.152	0.007	
		TS	Non‐OP	0.114	0.007	
			OP	0.122	0.008	
		PS	Non‐OP	0.019	0.008	
			OP	0.022	0.007	
	Y	WC	Non‐OP	0.174	0.024	< 0.0001**
			OP	−0.191	0.027	
		LR	Non‐OP	−0.005	0.005	0.006*
			OP	0.006	0.006	
		MS	Non‐OP	0.092	0.009	< 0.0001**
			OP	−0.09	0.010	
		TS	Non‐OP	0.082	0.009	< 0.0001**
			OP	−0.102	0.012	
		PS	Non‐OP	0.006	0.006	0.002*
			OP	−0.006	0.005	
	Z	WC	Non‐OP	−0.156	0.123	0.019*
			OP	−0.54	0.138	
		LR	Non‐OP	0.146	0.021	0.001*
			OP	0.031	0.035	
		MS	Non‐OP	−0.182	0.035	0.006*
			OP	−0.385	0.053	
		TS	Non‐OP	−0.152	0.064	0.050
			OP	−0.337	0.053	
		PS	Non‐OP	0.029	0.035	0.0006*
			OP	0.146	0.021	

*Note*: Sex as a fixed factor. Adjustment for multiple comparisons: Bonferroni. It should be noted that in Y direction (medial‐lateral), both sides have their impulses oppositely, but their magnitudes are similar, and thus the significant differences are ignored.

Abbreviations: Control: the control group with the same side as the THR side; LR: loading response; MS: mid‐stance; PS: pre‐swing phases; TS: terminal stance.

^a^ Covariates appearing in the model are evaluated at the following values: age = 55.69, BMI = 24.96, and mean speed = 1.29.

^b^ Covariates appearing in the model are evaluated at the following values: age = 60.76, BMI = 30.79, and mean speed = 1.04.

*p < 0.05 and **p < 0.01.

#### 3.3.2 Non‐operative Side Versus Control Side

**FIGURE 5:**
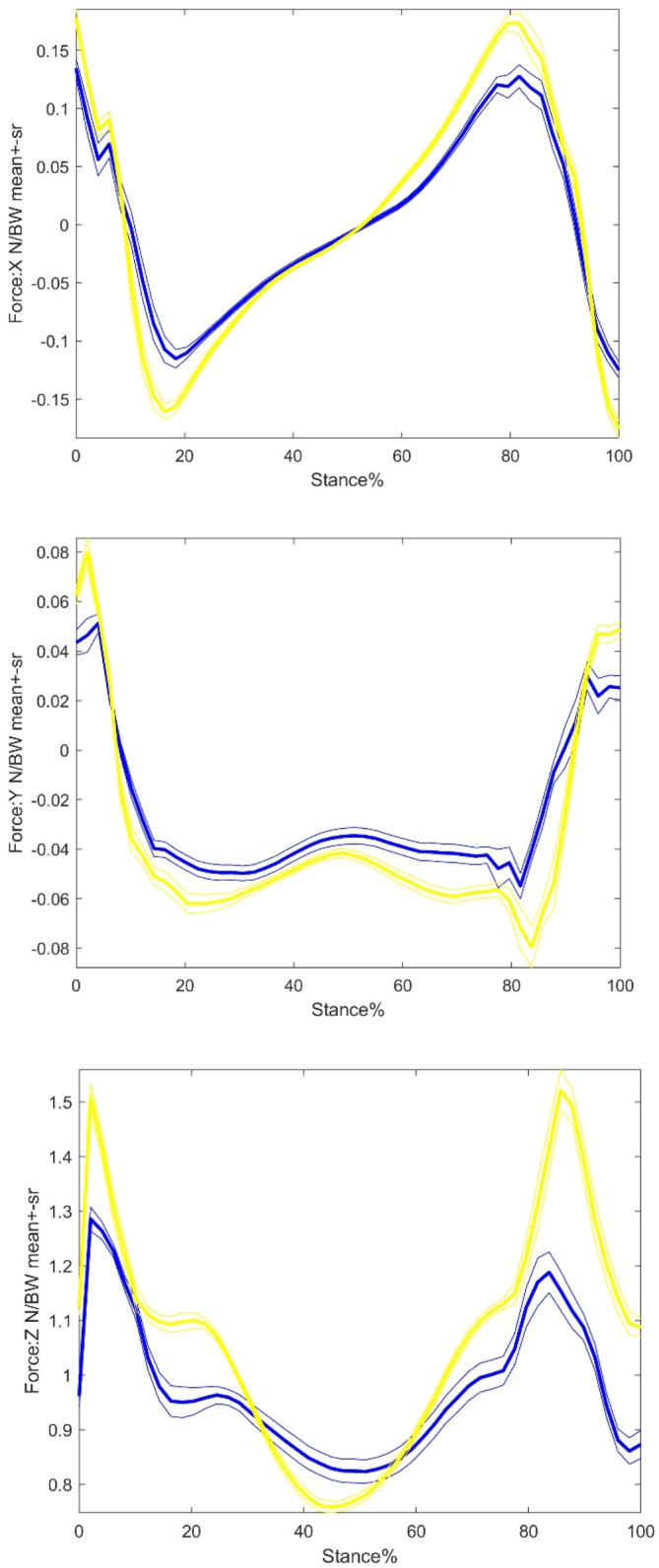
Comparison of the combined GRFs from both sides in stance phase between the control group (yellow) and the THR non‐operative side (blue). X, Y, and Z represent the anterior–posterior, medial–lateral and vertical directions. Yellow lines are the control and blue the THR non‐operative sides.

The combined GRFs in stance phase are shown in Figure 5 to compare the non‐operative side and control group side.

The statistical comparisons between the non‐operative and control sides are shown in Table 8. The main findings are that in vertical direction (Z), THR non‐operative side had lower impulse in PS than control group, but in the anterior–posterior direction (X), THR non‐operative side had larger impulse in PS than the control group, meaning that the non‐operative side had better push off or propulsion function.

#### 3.3.3 Comparison of Impulses Between Two Sides

The comparisons of impulses between paired sides in the THR are shown in Table 9. It should be noted that in the medial–lateral direction (Y), though the *p* values showed < 0.05, the reason is that both sides had opposite directions and the absolute values are similar, and thus there is no significant difference. The main findings are that in vertical direction (Z), THR operative side had lower impulse in LR than non‐operative side, but THR operative side had larger impulse in PS than the non‐operative side, meaning that the operative side had better push off but worse braking functions.

## 4 Discussion

### 4.1 Principal Findings

Regarding to specifically scientific hypotheses proposed in Introduction, the major discoveries are summarized as below. (1) the cadence, walking speed, stride length and step width in THR group were significantly decreased in compared with control group, while the stance and swing proportions were similar to the control group (Table 2); (2) the THR decreased loading response duration in operative side and pre‐swing duration in non‐operative side but increased mid‐stance duration in operative‐side and terminal‐stance in non‐operative side in compared with the control group, but the THR's two sides have similar duration proportions in sub‐stance phases (Tables 3 and 4); (3) the THR group had lower GRFs in the vertical direction than the control group but higher in the medial–lateral direction (Tables 5 and 6); (4) in operative side, the THR's impulses in loading response phase were lower than the control group in anterior–posterior and vertical directions (Table 7); (5) in non‐operative side, the THR's impulse in pre‐swing phase in anterior–posterior direction was higher than the control side (Table 8); and (6) in the vertical direction, THR's op‐side had lower impulses than the non‐op‐side in LR, MS and TS phases but higher in PS phases.

### 4.2 GRF

In the present study, it is found that THR participants showed a significantly lower level of Fz2 than healthy subjects, despite no clear difference in Fz1. It was found out in previous studies that both Fz1 and Fz2 were lower in the THR group [15, 16, 25]. However, walking speed was ignored in most of these studies. McCrory et al. [15] investigated the magnitude of vGRF (vertical GRF) in THR patients and counteracted the effect of walking speed on GRF by limiting the walking speed of the subjects. In this study, it is presumed that limiting the gait speed of a patient could not reflect their practical ability of daily walking. For this reason, walking speed was introduced as an interaction factor into the multivariable models to find out that only the THR subjects still displayed a significantly lower level of Fz2 on the operative side, suggesting that the reduction in Fz2 was not attributed solely to the low walking speed. Fz2 occurs when the heel leaves the ground, and the body produces a forward thrust to push the toes off the ground. The decrease in Fz2 indicates their inability to control the descent of the CoM effectively [26] and the failure to produce the upward force like the control, which leads to potential gait difficulties. This may be explained by the insufficient muscle strength around the remote joint in the lower leg [27, 28].

The Fx peak forces are reflective of the braking and propulsive abilities [29]. A reduction of Fxpp in the THR group was not observed in this study, which is unlike previous research results. Lunn et al. [30] discovered in a stratified study of age and walking speed that the braking and propulsive forces of THR patients declined compared to healthy people. In this study, this view is substantiated by factoring walking speed into the model. A lower propulsive force can have negative impact on gait speed, inter‐limb symmetry, and walking function [31], which also explains the low walking speed of THR patients in this study. On the one hand, lower propulsive force plays a central role in reduced walking speeds, while lower walking speed helps with posture control in gait, which may be seen as a protective factor for THR patients. On the other hand, there is a close correlation between walking speed and hip joint moment impulse, and there is a decrease in the adduction and total hip joint moment impulse with the increased gait [32]. Since the hip adduction moment impulse plays a significant role in generating the cumulative hip moments associated with the progression of hip OA, it's essential to increase the walking speed, which may be conducive to protecting the hip joint from degeneration. In the future, enhancing propulsive force deserves more attention for improving the patient's walking speed and it may be a therapeutic goal. Bowden et al. found out the close relationship between foot placement and the amount of propulsive impulse [33]. A lower step length was observed in the THR group than in the healthy group, which explains the reduction in Fx. One of the biomechanical reasons for the participants to limit Fx production in the targeted leg during gait lies in the inability to achieve adequate hip extension and reduced the active muscle forces, such as extensor hip muscle [31, 33]. Therefore, it is necessary to pay more attention to the patient's hip extensor muscle and hip joint extension range of motion during the follow‐up rehabilitation training.

To be our best knowledge, this study first found that the value of Fypp on the surgical side of THR patients was not significantly from that of the non‐surgical side. Previously, John et al. reported that the peak Fy increased sharply with walking speed [34], while in this study walking speed is factored into the model and find out a similar Fypp on the operative side of the THR patients to the non‐operative side, which indicates the balance between the operative and non‐operative sides of patients during walking, while significant difference of Fy peak forces observed between the THR group and the healthy group. At the later stage of support, the ground reaction force pushes the body inward, thus producing Fypp. For THR patients, the value of Fypp on the operative side was about 2 times higher than the control group, which indicated that the THR group walked with less stability than the control group and the patients need to pay higher forces in this direction than the control. [35]. THR patients may show reduced medio‐lateral stability as a result of the increased proprioception on their operative side, or the reliance on their contralateral limb for improved stability [36]. Gluteus medius muscles are the primary contributors to Fy, acting to rotate the body towards the swing leg during stance [37]. In the future, it is worth further exploring whether the difference in Fy between the legs of patients with THR is caused by the imbalance in muscle strength of the middle gluteal muscles between the two sides.

### 4.3 Impulse

In case of abrupt start or stop walking, there must be a rapid increase in anterior–posterior impulse. It was smaller in the THR group than in the healthy participants in loading response phases, which suggests a slight limit on the ability of THR patients to start in foot strike. The improvement of anterior–posterior impulse, especially the propulsive impulse, can be achieved by improving the ability of the ankle and knee muscle force through functional strength exercises [38]. A lower anterior–posterior impulse not only indicates the poor sensitivity of pace control but also suggests that THR subjects suffered from reduced load and shear force in the anterior–posterior direction of the lower limb joints [39, 40], which is beneficial to protect joints.

With respect to the vertical impulse, it has been demonstrated in previous studies that THR subjects showed a less significant vertical reaction force impulse than the control group [15]. In the present study, it is found that the changes of impulse in different walking phases were further investigated to obtain similar results. Interestingly, the differences in zIPs between the two groups varied significantly only in the loading response phase, and as whole cycle, zIP in the THR is lower than the control group, which may be attributed to the smaller Fz2. With none of the studies published to report these results on GRF and impulse of hip replacement subjects, it is difficult to compare our results with others.

In the present study, THR operative side exhibited smaller impulse in the anterior–posterior in the loading response phase which begins with the initial contact of the opposite limb and ends with ipsilateral toe, and THR non‐operative side had different impulses in later 3 sub‐stance phases from the control group. These results indicated that THR group used the non‐operative side to compensate the operative side. This compensation could help the operative side to recover the function. However, compensatory hip lifting movement is required for THR subjects to move the hip prosthesis, which causes THR subjects to consume three times more energy than healthy people [41]. Therefore, the affected limb may be unable to bear the sudden rise in energy consumption in the pre‐swing phase, thus showing abnormal 3D impulse changes. Therefore, postoperative rehabilitation is supposed to focus on the abnormal function of patients in the pre‐swing phase. In the medial–lateral direction, THR group had larger impulse than the control group in pre‐swing phase, which indicated instability. In the vertical direction, THR group had lower impulse than the control group in loading response and pre‐swing phases, meaning that the starting and ending in the stance were affected. Therefore, the THR group should pay more attention to their posture control and balance maintaining during the loading response and pre‐swing phases.

Although the recovery of lower extremity function among THR patients was not as satisfactory as that among the control group, there was no significant difference observed between the operative and non‐operative sides of patients in the THR group. It is indicated that the outcome of recovery is satisfactory on the operative‐side of patients in the THR group and a basically symmetrical force distribution on the non‐operative‐side is maintained during walking 1 year after surgery.

### 4.4 Limitation

There remain some limitations of this study, such as the small sample size for both the THR and control groups. It would be thus necessary to increase the sample size and include more matched control groups in further study. Also, surgical conditions have not been considered in THR group. Ideally, a THR group with a specific clinical condition was used so that the results would be more specific for the clinical service. In addition, the gender proportions and heights were not similar between the two groups. Though appropriate statistical methods were applied to reduce the effect of these factors on the results, future research with larger sample size and similar demographics from two groups is strongly recommended.

### 4.5 Advantages and Disadvantages

The advantage is that this study provides a preliminary attempt where using the impulses in 3D and sub‐stance phases interprets the differences between two groups; the detailed results and related information may help clinicians to design relevant exercise in rehabilitation for THR. The disadvantage is that the used methods require at least two force platforms and basic gait analysis equipment which may not be available in most hospitals. With the development of gait analysis technology, for example, marker less system to be applied, the disadvantage could be overcome in the future.

### 4.6 Future Study

Recently, there have been some studies using GRFs to different topics, for example, the center of forces used to explain the gait for the obesity group [2]; the body‐weight‐time integrated calculated to see if affected speed in terms of propulsion [42], the angular momentum used to analyze amputee gait [43]. Those studies have provided new ways to use GRFs for clinical practice. However, there is a lack of research on comparison between THR and control groups in terms of GRFs and impulses in three directions. As an attempt, this pilot study provides some initial results. In the future, this topic should be carried out using larger sample size than this study and also using other sets of GRF information [2, 42, 43].

## 5 Conclusions

The gait parameters, the durations, GRFs, and impulse in four phases of stance were compared between the THR and control groups during gait. It is found that (1) cadence, walking speed and stride length in THR group were decreased in compared with the control group; (2) the THR decreased LR duration in operative side and PS duration in non‐operative side in compared with the control group, but the THR's two sides have the similar duration proportions in sub‐stance phases; (3) the THR group had lower GRFs than the control group in vertical direction but higher in the medial–lateral direction; (4) in operative side the THR's impulses in loading response phases were lower than the control group in anterior–posterior direction, and (5) in non‐operative side, the THR's impulse in pre‐swing phase in anterior–posterior direction was higher than operative‐side and even higher than the control side. The findings from this study give useful reference to clinicians. A future study with larger sample size and similar demographics between two groups is highly recommended. Moreover, a study on a group of THR with a specific clinical condition would be recommended, as such a study could provide specific clinical plans in treatment and rehabilitation.

## Author Contributions

All authors had full access to the data in the study and take responsibility for the integrity of the data and the accuracy of the data analysis. Conceptualization, W.W.; Methodology, W.W. and Y.Z.; Investigation, Y.Z., W.R., G.A., and P.L.; Formal Analysis, Y.Z. and W.W.; Resources, W.R. and G.A.; Writing – Original Draft, Y.Z.; Writing – Review and Editing, W.W.; Visualization, W.W.; Supervision, W.W.; Funding Acquisition, W.W. and W.R.

## Ethics Statement

The study was conducted in accordance with the Declaration of Helsinki and approved by a local NHS research ethics committee (09/S1401/65) and the University of Dundee (EB/MC/LET/LN 1384).

## Consent

Informed consent was obtained from all subjects involved in the study.

## Conflicts of Interest

The authors declare no conflicts of interest.

## Data Availability

The data will be provided if a request to the corresponding author.
